# First-line treatment with infliximab versus conventional treatment in children with newly diagnosed moderate-to-severe Crohn’s disease: an open-label multicentre randomised controlled trial

**DOI:** 10.1136/gutjnl-2020-322339

**Published:** 2020-12-31

**Authors:** Maria M E Jongsma, Martine A Aardoom, Martinus A Cozijnsen, Merel van Pieterson, Tim de Meij, Michael Groeneweg, Obbe F Norbruis, Victorien M Wolters, Herbert M van Wering, Iva Hojsak, Kaija-Leena Kolho, Thalia Hummel, Janneke Stapelbroek, Cathelijne van der Feen, Patrick F van Rheenen, Michiel P van Wijk, Sarah T A Teklenburg-Roord, Marco W J Schreurs, Dimitris Rizopoulos, Michail Doukas, Johanna C Escher, Janneke N Samsom, Lissy de Ridder

**Affiliations:** 1 Paediatric Gastroenterology, Erasmus MC Sophia Children's Hospital, Rotterdam, The Netherlands; 2 Paediatric Gastroenterology, University Medical Center Amsterdam—Location VUmc, Amsterdam, The Netherlands; 3 Paediatrics, Maasstad Hospital, Rotterdam, The Netherlands; 4 Paediatrics, Isala Hospital, Zwolle, The Netherlands; 5 Paediatric Gastroenterology, Utrecht Medical Center/Wilhelmina Children's Hospital, Utrecht, The The Netherlands; 6 Paediatrics, Amphia Hospital, Breda, The Netherlands; 7 Referral centre for Paediatric Gastroenterology and Nutrition, Children's Hospital Zagreb, Zagreb, Croatia; 8 University JJ Strossmayer, School of Medicine Osijek, Osijek, Croatia; 9 Paediatric Gastroenterology, Children's Hospital, University of Tampere, Helsinki, Finland; 10 Tampere University, Tampere, Finland; 11 Paediatrics, Medical Spectrum Twente, Enschede, The Netherlands; 12 Paediatrics, Catharina Hospital, Eindhoven, The Netherlands; 13 Paediatrics, Jeroen Bosch Hospital, 's-Hertogenbosch, The Netherlands; 14 Paediatric Gastroenterology, University of Groningen, University Medical Centre Groningen, Groningen, The Netherlands; 15 Immunology, Erasmus MC, Rotterdam, The Netherlands; 16 Biostatistics, Erasmus MC, Rotterdam, The Netherlands; 17 Pathology, Erasmus MC, Rotterdam, The Netherlands; 18 Laboratory of Pediatrics, Division of Gastroenterology, Erasmus MC Sophia Children's Hospital, Rotterdam, The Netherlands

**Keywords:** inflammatory bowel disease, paediatric gastroenterology, IBD clinical, infliximab

## Abstract

**Objective:**

In newly diagnosed paediatric patients with moderate-to-severe Crohn’s disease (CD), infliximab (IFX) is initiated once exclusive enteral nutrition (EEN), corticosteroid and immunomodulator therapies have failed. We aimed to investigate whether starting first-line IFX (FL-IFX) is more effective to achieve and maintain remission than conventional treatment.

**Design:**

In this multicentre open-label randomised controlled trial, untreated patients with a new diagnosis of CD (3–17 years old, weighted Paediatric CD Activity Index score (wPCDAI) >40) were assigned to groups that received five infusions of 5 mg/kg IFX at weeks 0, 2, 6, 14 and 22 (FL-IFX), or EEN or oral prednisolone (1 mg/kg, maximum 40 mg) (conventional). The primary outcome was clinical remission on azathioprine, defined as a wPCDAI <12.5 at week 52, without need for treatment escalation, using intention-to-treat analysis.

**Results:**

100 patients were included, 50 in the FL-IFX group and 50 in the conventional group. Four patients did not receive treatment as per protocol. At week 10, a higher proportion of patients in the FL-IFX group than in the conventional group achieved clinical (59% vs 34%, respectively, p=0.021) and endoscopic remission (59% vs 17%, respectively, p=0.001). At week 52, the proportion of patients in clinical remission was not significantly different (p=0.421). However, 19/46 (41%) patients in the FL-IFX group were in clinical remission on azathioprine monotherapy without need for treatment escalation vs 7/48 (15%) in the conventional group (p=0.004).

**Conclusions:**

FL-IFX was superior to conventional treatment in achieving short-term clinical and endoscopic remission, and had greater likelihood of maintaining clinical remission at week 52 on azathioprine monotherapy.

**Trial registration number:**

ClinicalTrials.gov Registry (NCT02517684).

**Significance of this study**

**What is already known on this subject?**

- Crohn’s disease is an incurable, debilitating IBD that presents during childhood or adolescence in 8% of all patients with Crohn’s disease.
- In adult patients with Crohn’s disease, early infliximab (IFX) treatment has shown to affect the natural course of the disease, with a decrease in the occurrence of disease complications.
- Although corticosteroids are known to negatively impact growth and development in children and adolescents, IFX is currently reserved for corticosteroid and immunomodulator refractory paediatric Crohn’s disease.

**What are the new findings?**

- Ten weeks after start of therapy, the proportion of children in clinical and endoscopic remission is significantly higher in the group treated with first-line IFX than in the group that received exclusive enteral nutrition or prednisolone (conventional treatment).
- First-line IFX treatment is superior in achieving clinical remission on azathioprine monotherapy at 1 year without the need for corticosteroids or further biologics. A significant proportion of children in the conventional treatment group received an additional course of corticosteroids.
- Despite the possibility to escalate to treatment with IFX, the group that received conventional treatment shows poorer growth at 1 year.

**Significance of this study**

**How might it impact on clinical practice in the foreseeable future?**

- This study, being the first randomised controlled trial that investigates the effectiveness of first-line IFX in therapy-naïve paediatric patients with Crohn’s disease, argues that children with moderate-to-severe Crohn’s disease would benefit from first-line IFX treatment.
- Considering the detrimental impact of an insufficiently effective treatment strategy on growth, development and school attendance of these children and adolescents, a maximally effective therapy from diagnosis onwards is highly desired. This study provides evidence for starting IFX therapy in children with newly diagnosed moderate-to-severe Crohn’s disease.

## Introduction

In newly diagnosed paediatric patients with Crohn’s disease (CD), rapid disease control is desirable, but this outcome is not always achieved with current treatment strategies. The European Society of Paediatric Gastroenterology, Hepatology and Nutrition consensus guideline recommends starting with exclusive enteral nutrition (EEN) or oral corticosteroids for induction of remission in conjunction with immunomodulator maintenance treatment. Infliximab (IFX), an anti-tumour necrosis factor alpha (TNF-α) agent, is increasingly being used in paediatric patients with CD refractory to steroids and/or immunomodulators and results in high-sustained remission rates.^1^ IFX is started if response to the conventional treatment strategy case is inadequate.^2^

In many paediatric patients with CD, however, particularly in those with moderate-to-severe CD, mucosal healing and sustained clinical remission are not achieved with conventional treatment.^3^ First-line IFX (FL-IFX) is mentioned in the current paediatric CD treatment guidelines as the preferred strategy only for patients with CD with active perianal fistulising disease and those at risk of disabling disease.^2 4^ It has already been suggested by several observational studies, however, that primary IFX therapy may be very effective in inducing and maintaining clinical remission in paediatric patients with luminal CD.^5–7^ A randomised controlled trial (RCT) in adult patients with CD who had recently been diagnosed showed that early treatment with IFX in combination with immunomodulators was more effective than conventional treatment with corticosteroids, but an RCT in therapy-naïve patients has not been performed.^8^ As paediatric-onset CD often presents with a more severe phenotype of disease than adult-onset CD,^9^ this suggests that paediatric patients with CD may benefit even more from an FL-IFX strategy by preventing accumulating damage due to chronic uncontrolled inflammation.^10^ If mucosal healing can be achieved by establishing early control of inflammation, sustained clinical remission will be attained and development of complications such as strictures and perforations may be prevented in these paediatric patients.

We hypothesise that induction of remission with FL-IFX in moderate-to-severe paediatric patients with CD results in higher early clinical and endoscopic remission rates, and superior rate of clinical remission maintenance on azathioprine (AZA) monotherapy compared with conventional treatment. Therefore, we aim to compare the efficacy of FL-IFX treatment with conventional treatment in newly diagnosed patients with moderate-to-severe paediatric CD.

## Methods

### Study design and participants

We designed an investigator-initiated international open-label RCT in adherence to the Consolidated Standards of Reporting Trials statement. The trial was performed in 12 hospitals in three European countries (the Netherlands, Croatia and Finland). The study protocol was published.^11^ Inclusion and exclusion criteria are defined in table 1.

**Table 1:** Inclusion and exclusion criteria

Inclusion criteria	Exclusion criteria
Patient is 3–17 years of age	Indication for primary surgery
Patient presents with new-onset untreated CD according to the revised Porto criteria^34^	Symptomatic stenosis or stricture in the bowel due to scarring
wPCDAI >40 at baseline	Active perianal fistulas
Body weight >10 kg at baseline	Presence of a serious comorbidity, such as infection, sepsis, opportunistic infection, positive stool culture (*Salmonella enterica, Shigella* spp, *Yersinia enterocolitica* or *Campylobacter* spp), positive *Clostridium difficile* toxin assay or positive tuberculosis screening
	Presentation with suspected or definite pregnancy
	Already using CD-specific therapy

CD, Crohn’s disease; wPCDAI, weighted Paediatric Crohn’s Disease Activity Index.

It was aspired to enrol patients as soon as possible following diagnostic endoscopy. After CD diagnosis had been established and eligibility criteria had been met, written informed consent was obtained from the patient (if ≥12 years) and both parents and/or guardians.

### Randomisation and masking

Included patients were stratified by centre and equally randomised into two treatment groups with a validated variable block randomisation model, incorporated in the web-based database used for this trial (Castor Electronic Data Capture).^12^ Allocation was concealed for all participants and healthcare providers. Participants were randomly assigned to the experimental FL-IFX group or to the control group, referred to as the conventional treatment group. Participants, investigators and healthcare providers were not masked to treatment allocation.

### Procedures

**Figure 1:**
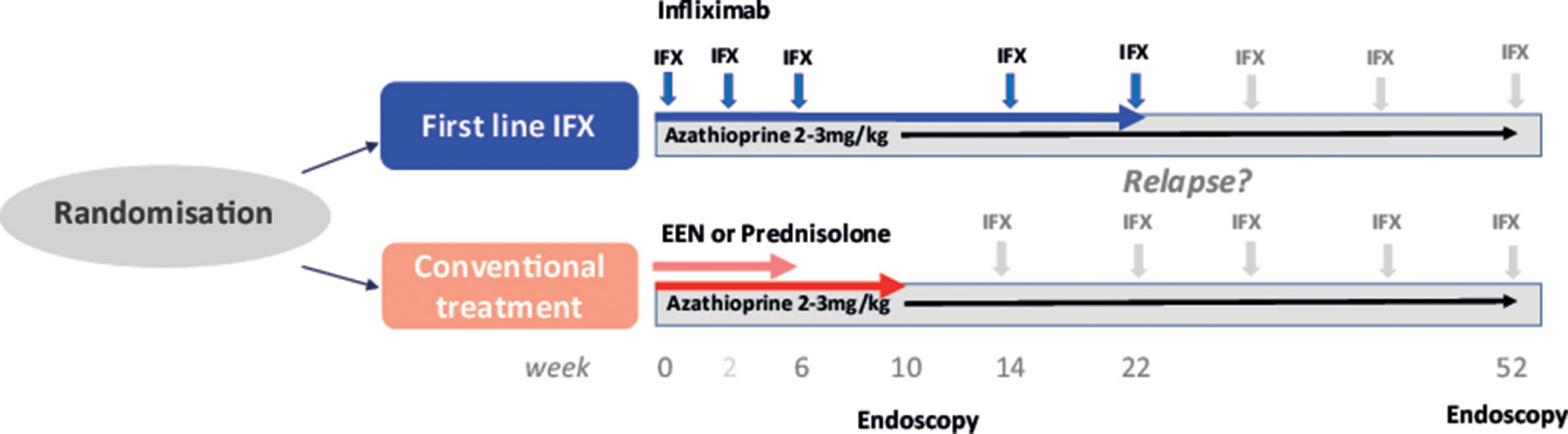
Trial design. Illustration of treatment procedures in this trial. EEN, exclusive enteral nutrition; IFX, infliximab.

The FL-IFX group received five intravenous IFX (Inflectra, CT-P13) infusions of 5 mg/kg induction at weeks 0, 2 and 6, followed by two maintenance infusions every 8 weeks. This was combined with oral AZA as maintenance treatment (once daily, dosed 2–3 mg/kg), which was initiated on the day induction treatment was started (figure 1). Conventional treatment consisted of standard induction treatment with either EEN (polymeric feeding for 6–8 weeks, after which normal diet was gradually reintroduced within 2–3 weeks) or oral prednisolone (for 4 weeks 1 mg/kg daily with a maximum of 40 mg, followed by tapering down to 5 mg per week until stop).^2^ Whether patients received induction treatment with EEN or prednisolone was based on patient preference, in accordance with the treating physician. Patients and parents were informed about all treatment options prior to randomisation. The choice between EEN and prednisolone was made after being assigned to the conventional treatment group. Similar to the FL-IFX group, both EEN and prednisolone were combined with oral AZA as maintenance treatment (2–3 mg/kg, once daily) in the conventional treatment group. AZA dosing was halved in case of thiopurine methyl transferase (TPMT) heterozygosity. As part of clinical care, AZA metabolites (6-thioguanine nucleotides and 6-methylmercaptopurine) were measured around the time of induction treatment cessation, and complete blood counts were performed weekly in the first month, monthly in the second and third months, and thereafter once every 3 months (online supplemental table 1). In both groups, methotrexate was the second choice immunomodulator, only prescribed in the event of low or absent TPMT activity or side effects of AZA.

10.1136/gutjnl-2020-322339.supp1 Supplementary data

In both groups, data were collected prior to start of induction therapy, at weeks 6, 10, 14, 22 and 52. At each visit, weighted Paediatric Crohn’s Disease Activity Index (wPCDAI) was determined,^13^ blood was obtained for routine laboratory analysis and serum samples were collected (in conventionally treated patients at start, week 10 and week 52). SD scores (SDS) adjusted for sex and age were used to evaluate linear growth. The height-for-age SDS were calculated with the Growth Analyser Research Calculation Tool, based on the Dutch national reference standards for all patients included in the Netherlands and the WHO growth reference standards for all patients included in other countries.^14^ Target height and target height SDS were calculated.^15^ Endoscopy (ileocolonoscopy) was performed prior to start of treatment, at week 10, and optionally at week 52. During endoscopy, the Simple Endoscopic Score for Crohn’s Disease (SES-CD) was used to evaluate endoscopic remission,^16^ which was defined as a SES-CD score <3. A single reader, blinded for both assigned treatment and time point, evaluated and rescored all endoscopic still images available by using the physician global assessment endoscopy score,^17^ to check interobserver variability between paediatric gastroenterologists (r=0.661, p>0.001). The SES-CD score was used for analyses regarding endoscopic findings. Faecal samples were collected for faecal calprotectin level measurement prior to start of treatment, at week 10 and at week 52. Faecal calprotectin levels were assessed in the Erasmus Medical Centre with ELISA (CALPRO assay). When faecal samples were missing, faecal calprotectin levels determined in the local hospital at this time point were used, which accounted for 15% of all samples. A faecal calprotectin level <100 µg/g was defined as biochemical remission.^18^ In patients ≥9 years old, quality of life (QOL) was assessed with the validated IMPACT III questionnaire. Scores range from 0 to 100, with a higher score indicating a better QOL.^19^

In case of non-response or absence of response (response being a decrease in wPCDAI of >17.5 points), the treatment advice for FL-IFX-treated patients was to shorten the IFX dosing interval to 6 weeks and/or to double the dose to 10 mg/kg. In accordance with clinical practice, clinicians could perform reactive therapeutic drug monitoring (TDM) to guide this decision. Initiation of IFX treatment was advised for conventionally treated patients. To guide clinical decision-making for treatment escalation, secondary loss of response was defined either by an increase of the wPCDAI with >17.5 points or by a total wPCDAI score >40 after response had been achieved. If FL-IFX-treated patients were not in clinical remission at week 22, it was recommended to continue the IFX infusions as standard care, instead of stopping after five infusions. Patients requiring such extended IFX therapy were considered treatment failures in intent-to-treat analysis of outcomes following five doses of FL-IFX. If FL-IFX-treated patients had loss of response during AZA monotherapy, it was advised to check AZA metabolite levels to assess optimal treatment. Contingent on optimal AZA metabolite levels, it was advised to restart IFX maintenance therapy every 8 weeks, also meaning treatment failure. Conventionally treated patients with loss of response during AZA monotherapy were advised to step up to IFX therapy after checking AZA metabolites and optimising its dosing in case of suboptimal levels. In addition to these guidelines, in patients without response, loss of response or intolerance to treatment, changes in treatment could be made according to the physician’s discretion.

### Outcomes

#### Primary outcome

The primary outcome of this study was clinical remission, defined as wPCDAI <12.5 at week 52, without need for treatment escalation. Any additional CD-related therapy or surgery during the 52 weeks was considered treatment escalation.

#### Definition of treatment escalation

Additional CD-related therapy in the FL-IFX group included (1) any course of corticosteroids, (2) increase of the IFX dose, (3) shortening of the IFX treatment interval, (4) continuation or restart of IFX after the standard five infusions, or (5) start of another biological agent. Additional CD-related therapy in the conventional treatment group included initiation of IFX and any course of corticosteroids that was additional to the standard treatment described in the previous section.

#### Secondary outcomes

Secondary outcomes included time-to-treatment escalation from start of induction and clinical disease activity scores over time. At week 10, clinical remission rate, endoscopic remission rate and faecal calprotectin level were assessed. QOL was evaluated at week 14. At week 52, the following outcomes were assessed: (1) additional corticosteroid use, (2) need for treatment escalation, (3) linear growth, (4) clinical remission rate, (5) endoscopic remission rate, (6) faecal calprotectin level, (7) QOL and (8) rate of adverse events. An adverse event was defined as any undesirable experience occurring to a subject during the study, whether or not it was considered to be related to the investigational product or the experimental treatment.

### Statistical analysis

Based on published studies reporting effectiveness of FL-IFX treatment and early IFX use^6^ in paediatric patients with CD, a power calculation was performed.^7 20^ Based on these studies a clinical remission rate of 60% in conventionally treated patients and 85% in FL-IFX-treated patients was expected. One-hundred patients (50 in each arm, considering a drop-out rate of 2%) were required to find this 25% difference in clinical remission at week 52 with a power of 80% (two-sided α 0.05).^11^ Data were analysed on an intention-to-treat basis. Safety analyses were based on the actual treatment patients received (ie, per protocol). Continuous variables were presented as medians and IQRs, and compared with the Mann-Whitney U test. Categorical variables were presented as absolute frequencies and percentages and compared by the X^2^ test or the Fisher exact test. The Wilcoxon signed rank test was used to compare height-for-age SDS at different time points within one treatment group. SES-CD scores with a missing ileum subscore due to the endoscopist’s failure to intubate the terminal ileum were included in the analysis to evaluate endoscopic remission. The median faecal calprotectin levels and SES-CD scores were subject to right censoring. To correct for this, medians of faecal calprotectin levels and SES-CD scores were calculated using the Kaplan-Meier method, and treatment groups for these outcomes were compared using the log-rank test. The multiple imputation method was used for missing erythrocyte sedimentation rate (ESR) levels (14.8%), missing albumin levels (10.5%) and missing faecal calprotectin levels (10.9%) in order to calculate biochemical remission rate. Twenty complete datasets were created for multiple imputation. For the primary outcome, no imputation was performed as <5% of data were missing. The time-to-treatment escalation outcomes were analysed using the Kaplan-Meier method. A paired analysis was performed for the linear growth. The mean clinical disease activity score over time was calculated with a linear mixed model, including the assigned treatment as a fixed effect and intercept as random effect. Random slopes were tested but not included.

All analyses were performed based on a significance level of 0.05. Calculations were performed using IBM SPSS Statistics V.24.0.

## Results

**Figure 2:**
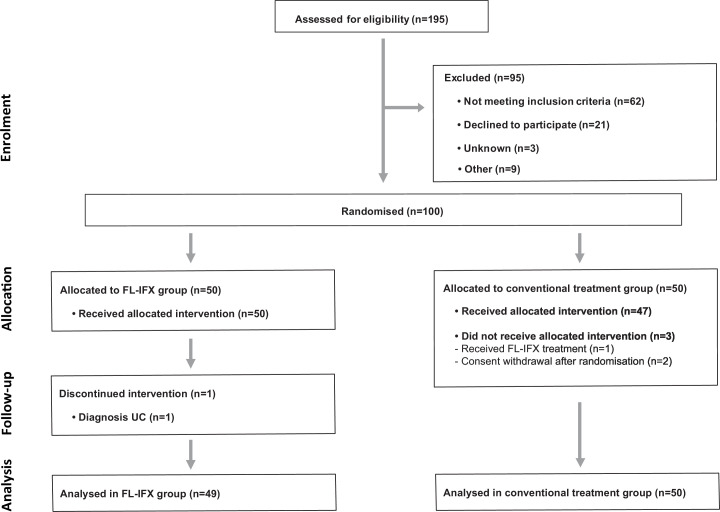
Trial profile. Flow chart of screened, randomised and treated patients. FL-IFX, first-line infliximab treatment.

Patients were recruited between 7 April 2015 and 19 November 2018. A total of 195 patients were screened for eligibility in this trial. One hundred patients were randomly assigned to FL-IFX (n=50) or conventional treatment (n=50) (figure 2). One patient in the conventional treatment group did not receive the study treatment she had been assigned to. Based on ethical considerations, she received the same (FL-IFX) treatment as her monozygotic twin sister, who had been included in this study previously. Two patients declined participation after randomisation, prior to the start of treatment. In the FL-IFX group, one patient was initially misclassified as CD, and this diagnosis was adjusted to ulcerative colitisat a later stage of the study. This patient, therefore, was excluded from all analyses. Patient and disease characteristics at baseline were similar between treatment groups (table 2).

**Table 2:** Baseline characteristics per treatment group

	FL-IFX (n=49)	Conventional (n=50)
Age at diagnosis (years)	15.1 (11.9–16.6)	14.1 (11.3–16.1)
Male sex (n)	24 (49.0%)	27 (54.0%)
Height (cm)	166 (154–175)	161 (143–170)
Height-for-age (SDS)	−0.07 (−0.84 to 0.76)	−0.53 (−1.06 to 0.26)
Weight (kg)	47.3 (36.8–57.1)	44.7 (30.3–55.0)
Tanner stage	4 (2–5)*	3 (1–4)*
wPCDAI	57.5 (47.5–67.5)	57.5 (47.5–73.8)
CRP (mg/L)	32.0 (11.5–46.5)	38.0 (22.0–65.9)
ESR (mm/hour)	35.0 (26.0–47.5)	32.0 (21.5–52.0)†
SES-CD	18.0 (11–26)†	18.0 (9–23)
Leucocytes (10^9^/L)	8.2 (7.3–10.7)	9.1 (6.8–11.9)
Faecal calprotectin (µg/g)	1114 (763–1869)	1086 (592–1661)
Perianal disease‡	5 (10%)	9 (18%)
Paris classification		
Age at diagnosis (years)		
<10	4 (8%)	9 (18%)
10–17	39 (80%)	37 (74%)
17–40	6 (12%)	4 (8%)
Disease location		
L1	12 (25%)	11 (22%)
L2	11 (22%)	12 (24%)
L3	25 (51%)	27 (54%)
Isolated L4	1 (2%)	–
Upper disease location		
No upper GI	29 (59%)	25 (50%)
L4a	19 (39%)	21 (42%)
L4b	1 (2%)	4 (8%)
Disease behaviour		
B1	46 (94%)	43 (86%)
B2	3 (6%)	7 (14%)
B3	–	–
B2B3	–	–
Growth delay	0 (0%)†	2 (4%)
Time between diagnostic endoscopy and start of treatment (days)	9 (5–14)	7 (2–14)

Data are presented as n (%) or median (IQR).

Baseline characteristics were not significantly different between treatment groups.

*>1 missing data point.

†One missing data point.

‡Perianal disease comprised inactive fistula, skin tags or anal fissures.

CRP, C reactive protein; ESR, erythrocyte sedimentation rate; FL-IFX, first-line infliximab; SDS, SD score; SES-CD, Simple Endoscopic Score for Crohn’s Disease; wPCDAI, weighted Paediatric Crohn’s Disease Activity Index.

The median time between diagnostic endoscopy and start of treatment for all included patients was 8 days (IQR 4–14). Twenty-seven patients (56%) in the conventional treatment group received EEN as primary induction therapy, while 20 patients (42%) received prednisolone (online supplemental table 2A).

### Efficacy of induction therapy

Ten weeks after start of induction therapy, significantly more FL-IFX-treated patients than conventionally treated patients were in clinical remission (59% (24/41) vs 34% (15/44), p=0.021). Fifty-seven patients (27 FL-IFX and 30 conventional), with similar baseline characteristics (online supplemental table 2B), underwent endoscopy at week 10. A higher proportion of patients in the FL-IFX group achieved endoscopic remission (16/27 (59%) vs 5/30 (17%), p=0.001, table 3) and median SES-CD scores were lower in the FL-IFX group (3 (IQR 0–5) vs 9 (IQR 3–19), p=0.005). In addition, the proportion of patients with a faecal calprotectin level <100 µg/g was higher in the FL-IFX group, and C reactive protein, ESR and leucocyte levels were lower (table 3).

**Table 3:** Findings at 10 weeks after start of induction therapy in the first-line IFX group versus the conventional treatment group

	First-line IFX	Conventional	P value
Fcal (µg/g), median (IQR)	286 (62–596)	545 (279–1108)	0.004
Patients with Fcal <100 µg/g, n (%)	13/39 (33)	5/38 (13)	0.036
CRP (mg/L), median (IQR)	2.0 (0.8–3.2)	8.4 (2.0–23.8)	<0.001
ESR (mm/hour), median (IQR)	6.5 (3.0–17.3)	17 (8.0–33.0)	0.003
Total leucocyte count (10^9^/L), median (IQR)	5.5 (4.8–7.1)	7.3 (5.9–9.3)	0.001

CRP, C reactive protein; ESR, erythrocyte sedimentation rate; Fcal, faecal calprotectin; IFX, infliximab.

### Treatment course

**Figure 3:**
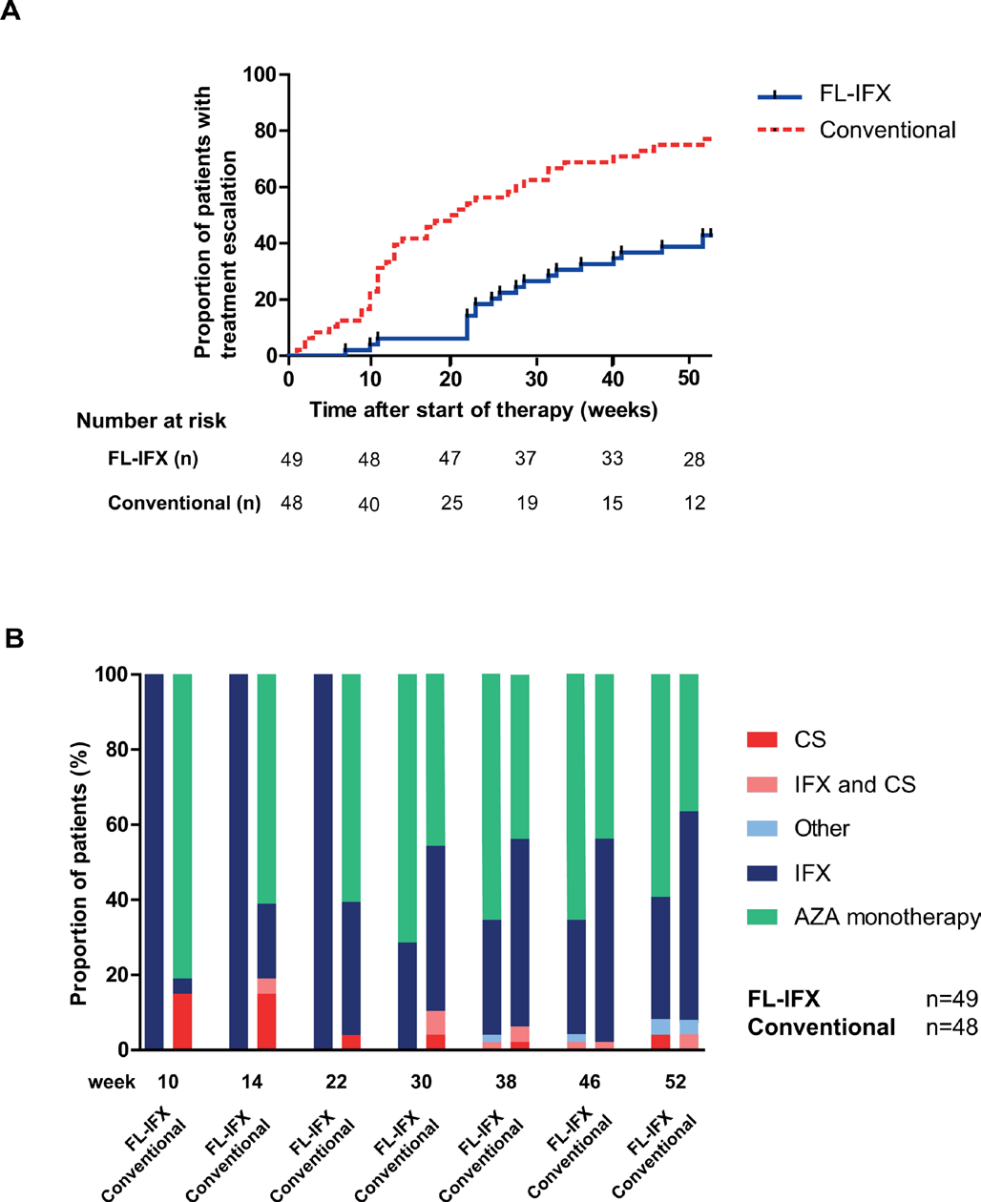
Proportion of patients who needed treatment escalation. (A) Kaplan-Meier estimates of the time-to-treatment escalation after start of therapy. Any additional CD-related therapy or surgery during the 52 weeks was considered treatment escalation. Additional CD-related therapy in the FL-IFX group included: (1) any course of corticosteroids, (2) increase of the IFX dose, (3) shortening of the IFX treatment interval, (4) continuation or restart of IFX after the standard five infusions or (5) start of another biological agent. In the conventional treatment group, additional CD-related therapy included start of IFX and any course of corticosteroids that was additional to the standard treatment. (B) Proportion of patients receiving each treatment option from 10 weeks onwards, depicted per randomised group. AZA, azathioprine; CD, Crohn’s disease; CS, corticosteroid; FL-IFX, first-line infliximab.

The mean wPCDAI score at all time points, corrected for repeated measurements, was lower in the FL-IFX group than in the conventional treatment group, although not significantly different (9.8 vs 14.2, respectively, p=0.07) (online supplemental figure 1). During the 52 weeks of follow-up, 43% of patients (95% CI 30% to 57%) in the FL-IFX group and 75% of patients (95% CI 60% to 86%) in the conventional treatment group needed treatment escalation (p=0.001, figure 3A). Disease activity scores and level of inflammatory markers at 10 and 14 weeks after induction treatment were higher in those who received treatment escalation than in those who did not (online supplemental table 3).

10.1136/gutjnl-2020-322339.supp2 Supplementary data

10.1136/gutjnl-2020-322339.supp3 Supplementary data

#### FL-IFX treatment

Twenty-one patients in the FL-IFX group needed treatment escalation. Twelve (24.5%) continued IFX therapy after the five per-protocol infusions (table 4A and figure 3B). Twenty-eight patients did not need treatment escalation.

**Table 4A:** Type of treatment escalation in the FL-IFX treatment group within 52 weeks

Number of patients needing treatment escalation in the FL-IFX group, per-treatment escalation type	
Continuation of IFX after five infusions; n (%)	12/49 (24.5)
Restart anti-TNF therapy; n (%)	7/49 (14.5)
Infliximab; n	6
Adalimumab; n	1
Corticosteroid course; n (%)	2/49 (4)

FL-IFX, first-line infliximab; TNF, tumour necrosis factor.

Based on reactive TDM 2/49 patients received dose escalation within the first 22 weeks. None of the seven patients that restarted IFX experienced side effects or needed to stop within the first year of follow-up.

#### Conventional treatment

As depicted in figure 3B, 40% of conventionally treated patients were already escalated to a second course of corticosteroids (19%) or IFX (21%) at week 14. Twenty patients received one or more courses of corticosteroids on top of the per-protocol use within the first year (table 4B). This resulted in extra corticosteroid use for a median duration of 67 days (IQR 53.3–72.3) in these patients. None of the patients received an extra EEN course. Thirty-six patients in the conventional treatment group needed treatment escalation.

**Table 4B:** Type of treatment escalation in the conventional treatment group

Number of patients needing treatment escalation in the conventional group, per-treatment escalation type	
Intensification to IFX; n (%)	16/48 (33)
Additional corticosteroids followed by IFX; n (%)	13/48 (27)
One or more courses of corticosteroids; n (%)	7/48 (15)

IFX, infliximab.

### Findings after 1-year follow-up

**Figure 4:**
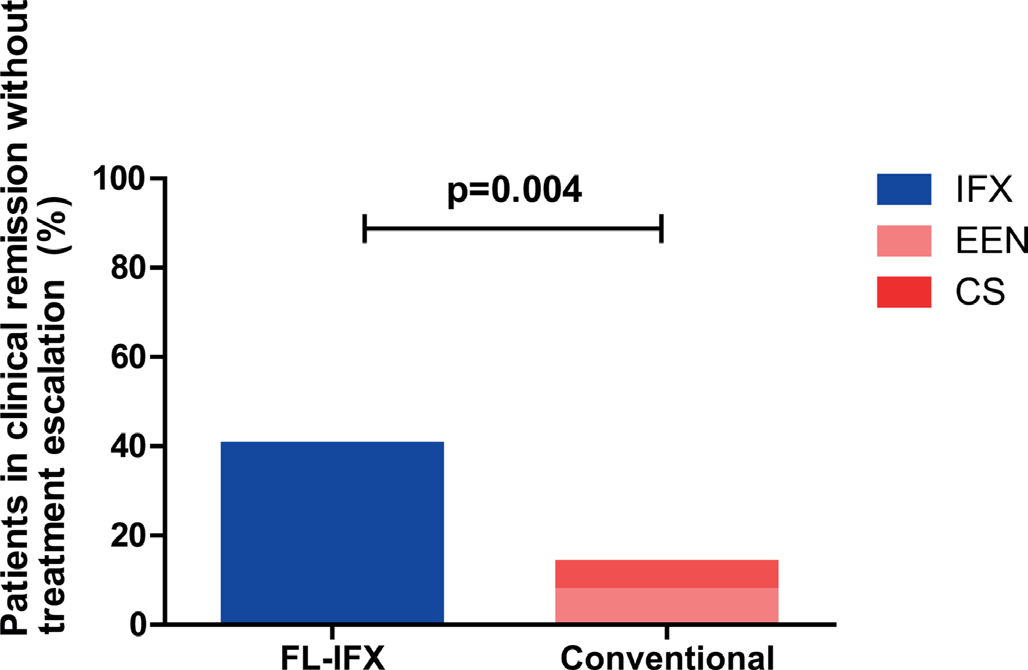
Proportion of patients in clinical remission without treatment escalation. The proportion of patients in clinical remission, defined as a weighted Paediatric Crohn’s Disease Activity Index <12.5, without treatment escalation at 52 weeks after the start of induction therapy. CS, corticosteroid; EEN, exclusive enteral nutrition; FL-IFX, first-line infliximab.

At week 52, the primary outcome of clinical remission without need for treatment escalation was reached in more FL-IFX-treated patients than in conventionally treated patients. In particular, 19/46 (41%) of the FL-IFX-treated patients were in clinical remission without need for treatment escalation, vs 7/48 (15%) of the conventionally treated patients (figure 4). This resulted in a 26% absolute difference (95% CI 0.18% to 0.35%, p=0.004). Irrespective of any treatment escalation during the study period, no significant differences were found in clinical, biochemical and endoscopic remission at week 52 (table 5).

**Table 5:** Findings at week 52 per treatment group

	First-line IFX	Conventional	P value
wPCDAI, median (IQR)	7.5 (0–15)	10 (0–17.5)	0.476
Clinical remission, n (%)	33/47 (70)	26/46 (57)	0.420
Clinical remission in patients on immunomodulator monotherapy, n (%)	22/29 (76)	12/18 (67)	0.958
Endoscopic remission, n (%)*	5/18 (28)	5/14 (36)	0.630
SES-CD, median (IQR)	7 (2–7)	6 (0–10)	0.961
Fcal <100 µg/g, n (%)	17/48 (35)	9/47 (19)	0.120

Clinical remission is defined as a wPCDAI <12.5. Endoscopic remission was defined as a SES-CD <3. The group of patients on immunomodulator monotherapy comprised patients on azathioprine (n=46) and methotrexate (n=1). Baseline characteristics of these patients are similar (online supplemental table 2C).

*Eighteen FL-IFX patients and 14 conventionally treated patients consented for endoscopy at week 52.

Fcal, faecal calprotectin; IFX, infliximab; SES-CD, Simple Endoscopic Score for Crohn’s Disease; wPCDAI, weighted Paediatric Crohn’s Disease Activity Index.

In contrast, in the FL-IFX-treated patients median SDS height-for-age significantly improved between baseline and week 52 (median SDS of −0.07 (IQR −0.84 to 0.76) at baseline vs 0.02 (IQR −0.81 to 0.70) at week 52, p=0.045), while it significantly decreased in conventionally treated patients (median SDS of −0.53 (IQR −1.06 to 0.26) at baseline vs −0.66 (IQR −1.13 to 0.11) at week 52, p=0.020) (table 6).

**Table 6:** Change in SDS height-for-age between baseline and 52 weeks

	First-line IFX (n=48)	Conventional (n=47)	P value
SDS height for age at baseline, median (IQR)	−0.07 (−0.84 to 0.76)	−0.53 (−1.06 to 0.26)	0.069
SDS height for age at week 52, median (IQR)	0.02 (−0.81 to 0.70)	−0.66 (−1.13 to 0.11)	0.021
Change in SDS height-for-age between baseline and 52 weeks	0.08 (−0.05 to 0.21)	−0.08 (−0.23 to 0.04)	0.002
Median increase in height (cm) between baseline and 52 weeks	4.0 (1.1–6.2)	2.4 (0.7–5.4)	0.226

IFX, infliximab; SDS, SD score.

### Quality of life

At week 14 and week 52, the median QOL scores in the FL-IFX group and conventional group were similar and in both groups significantly higher at both time points than at baseline (online supplemental table 4). The median QOL score in the FL-IFX group increased from 59.3 at baseline (IQR 48.2–71.8) to 79.7 at week 52 (IQR 70.9–88.5, p<0.001) and in the conventional group from 61.2 (IQR 49.8–70.7) to 77.5 (IQR 66.3–85.0, p<0.001).

### Safety

There was no significant difference between the proportion of patients with an adverse event in the FL-IFX group (44%) versus the conventional treatment group (60%; absolute difference of 16%; 95% CI: −0.04% to 0.33%, p=0.125). In total, 94 adverse events occurred, 40 of which were reported in FL-IFX-treated patients and 54 in conventionally treated patients. Fifteen serious adverse events were reported (table 7).

**Table 7:** Reported serious adverse events during 52 weeks of follow-up

	First-line IFX (n=50)	Conventional (n=47)	Total
Ileocecal resection	1*	2*,^*^	3
Intra-abdominal abscess	1*	1*	2
Psychosis	1*	0	1
Perianal abscess drainage	0	3*‡‡	3
Excision of pilonidal cyst	1†	0	1
Hospitalisation	2*,^*^	3*‡,‡	5
Total	**6**	**9**	**15**

*One patient treated with IFX and azathioprine.

†One patient treated with azathioprine.

‡One patient treated with prednisolone and azathioprine.

IFX, infliximab;

## Discussion

This is the first RCT to compare the efficacy of IFX directly after diagnosis to conventional treatment with corticosteroids or EEN in newly diagnosed paediatric patients with moderate-to-severe CD. Ten weeks after induction therapy, higher clinical remission rates are found in the FL-IFX group. Of the FL-IFX-treated patients that underwent an endoscopy at week 10, a higher proportion is in endoscopic remission than in the conventional treatment group. Overall, the proportion of patients in clinical and biochemical remission at 1 year did not significantly differ between the two treatment groups. However, the trajectory towards remission is very different between groups. In particular, FL-IFX treatment is superior to conventional treatment in achieving clinical remission without need for treatment escalation 1 year after the start of therapy. Children with moderate-to-severe CD benefit from an effective therapy from diagnosis onwards. In this young population, delay in achieving remission and frequent flare-ups in the first year after diagnosis may slow their pubertal development and affect their school attendance and general well-being.^21^ Moreover, ineffective induction treatment strategies in children and adolescents put them at risk of developing fistulising or stricturing complications.^9^ Findings from the GROWTH Study show that children with higher inflammatory markers after induction treatment were more at risk of a disease relapse in 18 months and early surgery.^22 23^ Similarly, we found in our cohort that inflammatory markers after induction treatment were higher in patients needing treatment escalation. Frequent or ongoing corticosteroid use, which is needed in 42% of the conventionally treated group in our cohort, has debilitating side effects and may also affect growth. Our finding, that the SDS height-for-age decreases in significantly more conventionally treated patients than FL-IFX-treated patients during the first year, argues that conventional treatment provides insufficient disease control. In addition, steroid-sparing therapy may also result in a lower chance of developing disease complications.^24^ While the efficacy of IFX in refractory paediatric patients with CD is well established,^1 2^ this RCT now proves what was suggested in only a small number of observational cohort studies in children with CD: that FL-IFX therapy results in lower relapse rate and longer duration of remission than induction with EEN or corticosteroids.^5 6 20 25^

In our cohort, endoscopic remission rates in FL-IFX-treated patients were significantly higher at week 10 than those in conventionally treated patients. The endoscopic remission rate of 59% in the FL-IFX group is superior to previously reported endoscopic remission rates in both paediatric and adult studies,^26 27^ which may be explained by the primary IFX use in our study versus the secondary use of IFX in other paediatric studies.^28^ Clinical and endoscopic remission rates in the conventionally treated group are lower than previously shown by Borrelli *et al*.^3^ This difference may be due to the use of a stricter definition of endoscopic remission (SES-CD <3) in our cohort and the 2–4 weeks’ longer duration of EEN in the Italian cohort. It could suggest that children with moderate-to-severe CD may benefit from EEN treatment with a duration of more than 6 weeks. The majority of conventionally treated patients in our study did not reach clinical remission without the need for additional therapies. However, if the 15% of patients in the conventional treatment group that did achieve clinical remission without treatment escalation would have received FL-IFX, they might have been overtreated. As we have so far been unable to discriminate these patients on the basis of their clinical profile at diagnosis, studies identifying predictors of disease course and treatment response are essential.

Since the design of this study in 2015, guidelines were updated and the role of therapeutic drug monitoring has increased. In our study, reactive TDM was performed based on clinical practice and thus the clinician’s decision. In only a few patients this resulted in treatment optimisation, as only 2 out of 49 patients received interval shortening and/or dose escalation within the first 22 weeks. Thus far, IFX could be safely restarted in the patients in our cohort. However, the risk of increased immunogenicity and consecutive loss of response after restarting IFX has been demonstrated and longer follow-up of our cohort is needed.^29^ Based on progressive understanding in clinical practice since the design of our study, we do not favour stopping FL-IFX therapy after five infusions. However, in adult patients with IBD, the concept of cycles of biologics treatment and planned de-escalation is currently being investigated. Reenaers *et al* demonstrated that retreatment with IFX was effective and well-tolerated in a group of patients that stopped IFX treatment after at least 1 year and 6 additional months of corticosteroid-free remission.^30^ In 60% of FL-IFX-treated patients in our cohort, there was no need to continue or restart IFX within 6 months after the fifth infusion; they continued on AZA monotherapy. Concerns have been raised about the use of AZA maintenance therapy, especially due to the associated risk of lymphoproliferative disorders.^31^ Although international guidelines and clinical practice differ regarding the use of AZA in CD,^2 4^ this may be a reason to continue IFX monotherapy after five infusions instead of continuing AZA monotherapy. We cannot draw firm conclusions which treatment strategy is most effective and safe as our study was not designed to investigate the effectiveness of IFX monotherapy versus AZA monotherapy after five IFX infusions.

Counterarguments for implementation of FL-IFX therapy could be the increased risk of side effects and higher costs.^32^ The overall incidence of adverse events within 1 year was similar between both treatment groups, which is in line with findings in adults^7^ and indicates that the use of first-line anti-TNF in these patients is safe. The introduction of biosimilars has led to significantly decreased costs of IFX treatment.^33^ In cost-effectiveness studies patients received the originator IFX, whereas children in our study received the biosimilar CT-P13.^32^

A clear strength and innovative aspect of this study is the inclusion of new-onset and therapy-naïve patients with CD. Performing an RCT in children with CD is rarely done and extremely challenging. Only 21 patients and their parents declined to participate in this trial, which demonstrates the patients’ interest in FL-IFX treatment. There are some drawbacks associated with our study. First, treatment assignment and assessments guiding treatment changes were not masked for patients and investigators, which could have created a performance bias. However, this bias is partly mitigated by the evaluation of growth, faecal calprotectin and endoscopic remission as objective outcome measures in this study. Second, while participating in the study, not all patients agreed on the endoscopic evaluation scheduled in this study at weeks 10 and 52. Based on ethical considerations, these patients continued to participate in the study, which led to missing endoscopic results. Despite the lower numbers, this did not introduce a bias as patients who underwent endoscopy had comparable disease characteristics at baseline (online supplemental table 2B, C). Third, the difference in duration of therapy between FL-IFX (five IFX infusions in 22 weeks) and conventional treatment (6–8 weeks of EEN or 10 weeks of prednisolone) may have influenced the interpretation of our findings. The duration of conventional treatment, however, was in accordance with paediatric CD guidelines and, as such, reflects current clinical practice.^2^ We expect the effect of the difference in therapy duration to be minimal, as both groups had received AZA monotherapy per protocol for at least 6 months at 52 weeks. Although AZA metabolite levels were measured as part of clinical care in 74/97 patients and were therapeutic on average, we did not incorporate these results in our conclusions as data were collected in a non-standardised fashion.

In conclusion, despite the similar clinical remission rates at 1 year after diagnosis in both treatment groups, we argue that children and adolescents with moderate-to-severe CD would benefit from FL-IFX treatment as an insufficiently effective treatment strategy impacts their growth and development. Ongoing disease activity or corticosteroids use prior to escalation to IFX in the conventional treatment group could have been prevented by starting FL-IFX. This innovative treatment was well accepted by children and their parents, which shows the importance of moving forward with protocols to allow us to learn what is best. Future follow-up and additional research are needed to determine whether IFX can be stopped and for which patients this will be beneficial.

## Data Availability

No data are available. The data that support the findings of this study are available from the corresponding author, upon reasonable request.
