# Supplementary material for: First-line treatment with infliximab versus conventional treatment in children with newly diagnosed moderate-to-severe Crohn’s disease: an open-label multicentre randomised controlled trial
Source: Gut. 2020 Dec 31;71(1):34–42. doi: 10.1136/gutjnl-2020-322339 (PMC8666701; doi:10.1136/gutjnl-2020-322339)
Supplement: Supplementary data [file gutjnl-2020-322339supp001.pdf]

SUPPLEMENTAL TABLES

	First-line IFX n=35	Conventional n=39	P-value
**6-MMP levels**	1400 (800-2700)	1500 (597-2875)*	0.818
**6-TGN levels**	567 (438-660)	490 (224-650)	0.258
**6-MMP/6-TGN ratio**	3.21 (1.57-5.38)	2.91 (1.25-6.35)*	0.881

Supplemental Table 1. Azathioprine metabolites measured according to clinical practice. *Three out of 29 6MMP levels were missing. IFX=infliximab; 6 MMP=6-methylmercaptopurine; 6 TGN=6-thioguanine nucleotide.

		Exclusive enteral nutrition (n=27)	Oral prednisolone (n=20)	P value
**Age at diagnosis, years**		14.5 (12.4-16.5)	13.8 (11.6-15.6)	0.282
**Male sex**		16 (59%)	10 (50%)	0.528
**Height, cm**		164.3 (149.2-175.0)	157.4 (143.5-16.6)	0.169
**Weight, kg**		48.5 (33.8- 56.3)	41.8 (31.1-53.9)	0.240
**wPCDAI**		60.0 (48.8-67.8)	53.8 (45.6-81.3)	0.909
**CRP, mg/L**		34.5 (24.7-65.9)	39.5 (20.5-69.0)	0.921
**ESR, mm/hour**		27.0 (17.0-47.5)	34.0 (22.5-71.0)	0.091
**SES-CD**		15 (6-22)	19 (6-28)	0.275
**Leukocytes, 10^9/L**		8.9 (6.8-11.7)	9.1 (6.2-11.8)	0.982
**Faecal calprotectin, µg/g**		1197 (1033-1661)	592 (555-1133)	0.014
**Perianal disease†**		5 (19%)	4 (20%)	0.898
**Paris classification**				
**Age at diagnosis**	<10 years	4 (15%)	3 (15%)	0.757
	10-17 years	20 (74%)	16 (80%)	
	17-40 years	3 (11%)	1 (5%)	
**Disease location**	L1	6 (22%)	5 (25%)	0.755
	L2	6 (22%)	6 (35%)	
	L3	15 (56%)	9 (45%)	
	Isolated L4	-	-	
**Upper disease location**	No upper GI	14 (52%)	11 (55%)	0.942
	L4a	12 (44%)	8 (45%)	
	L4b	1 (4%)	1 (5%)	
**Disease behavior**	B1	25 (93%)	16 (80%)	0.201
	B2	2 (7%)	4 (20%)	
	B3	-	-	
	B2B3	-	-	
**Growth delay**		1 (4%)	1 (5%)	0.828
**Time between diagnostic endoscopy and start of treatment, days**		7 (1-13)	8 (3-17)	0.266

Supplemental Table 2A: Baseline characteristics of patients treated with exclusive enteral nutrition versus oral prednisolone. Data are presented as n (%) or median (IQR)**.** wPCDAI, weighted paediatric Crohn's disease activity index; CRP, C-reactive protein; ESR, erythrocyte sedimentation rate; SES-CD, Simple Endoscopic Score for Crohn's Disease; †perianal disease comprised inactive fistula, skin tags, or anal fissures**.**

		FL-IFX (n=27)	Conventional (n=30)	P value
**Age at diagnosis, years**		15.1 (11.9-16.6)	14.1 (11.3-16.1)	0.181
**Male sex**		15 (49%)	18 (54%)	0.734
**Height, cm**		168 (151-177)	161 (143-174)	0.136
**Weight, kg**		52.0 (34.0-63.0)	43.2 (28.8-55.0)	0.248
**wPCDAI**		57.5 (47.5-65)	50 (45-75.6)	0.937
**CRP, mg/L**		29.0 (12.0-46.0)	39.5 (22.0-65.9)	0.091
**ESR, mm/hour**		35.0 (27.0-55.0)	28.0 (18.0-60.0)*	0.795
**SES-CD**		19 (9-24)	17 (6-19)	0.869
**Leukocytes, 10^9/L**		8.6 (7.4-10.4)	9.3 (7.4-12.1)	0.895
**Faecal calprotectin, µg/g**		1045 (684-1228.0)	1078 (617-1661)	0.765
**Perianal disease†**		2 (7%)	7 (23%)	0.100
**Paris classification**				
**Age at diagnosis**	<10 years	2 (7.5%)	5 (17%)	0.568
	10-17 years	23 (75%)	23 (76%)	
	17-40 years	2 (7.5%)	2 (7%)	
**Disease location**	L1	8 (30%)	5 (17%)	0.392
	L2	5 (18%)	9 (30%)	
	L3	13 (48%)	16 (53%)	
	Isolated L4	1 (4%)	-	
**Upper disease location**	No upper GI	12 (44%)	16 (53%)	0.252
	L4a	15 (56%)	12 (40%)	
	L4b	-	2 (7%)	
**Disease behavior**	B1	25 (92%)	26 (87%)	0.467
	B2	2 (8%)	4 (13%)	
	B3	-		
	B2B3	-	-	
**Growth delay**		0 (0%)	2 (7%)	0.172
**Treated with exclusive enteral nutrition**			16 (53%)	
**Time between diagnostic endoscopy and start of treatment, days**		9 (6-13)	8.5 (2-14.5)	0.860

Supplemental Table 2B: Baseline characteristics of patients that underwent an endoscopic evaluation at week 10. Data are presented as n (%) or median (IQR)**.** FL-IFX, first-line IFX; wPCDAI, weighted paediatric Crohn's disease activity index; CRP, C-reactive protein; ESR, erythrocyte sedimentation rate; SES-CD, Simple Endoscopic Score for Crohn's Disease; EEN, exclusive enteral nutrition**.** *1 missing data point**.** †perianal disease comprised inactive fistula, skin tags, or anal fissures**.**

		FL-IFX (n=18)	Conventional (n=14)	P value
**Age at diagnosis, years**		15.5 (11.1-16.6)	12.0 (9.4-14.6)	0.168
**Male sex**		10 (55.6%)	8 (57.1%)	0.928
**Height, cm**		160.9 (146.4-174.3)	147.9 (135.0-167.6)	0.193
**Weight, kg**		45.6 (31.3-55.5)	36.3 (24.9-43.0)	0.338
**wPCDAI**		56.3 (49.4-73.1)	52.5 (45.0-61.9)	0.338
**CRP, mg/L**		28.5 (7.8-40.3)	27.0 (17.8-46.0)	0.168
**ESR, mm/hour**		39.0 (30.0-40.0)	29.5 (19.8-62.5)	0.694
**SES-CD**		16 (10-23)	15 (8-15)	0.346
**Leukocytes, 10^9/L**		8.6 (7.4-10.4)	7.5 (5.4-11.7)	
**Faecal calprotectin, µg/g**		1076 (652-1445)	1033 (555-1087)	0.337
**Perianal disease†**		2 (6%)	7 (23%)	0.109
**Paris classification**				
**Age at diagnosis**	<10 years	3 (17%)	5 (36%)	0.250
	10-17 years	13 (72%)	9 (64%)	
	17-40 years	2 (11%)	-	
**Disease location**	L1	4 (22%)	4 (28.5%)	0.773
	L2	4 (22%)	4 (28.5%)	
	L3	9 (50%)	6 (49%)	
	Isolated L4	1 (6%)	-	
**Upper disease location**	No upper GI	9 (50%)	11 (78%)	0.073
	L4a	9 (50%)	2 (14%)	
	L4b	-	1 (7%)	
**Disease behavior**	B1	17 (94%)	10 (71%)	0.075
	B2	1 (6%)	4 (29%)	
	B3	-	-	
	B2B3	-	-	
**Growth delay**		0 (0%)	1 (7%)	0.249
**Treated with exclusive enteral nutrition**			7 (50%)	
**Time between diagnostic endoscopy and start of treatment, days**		10 (4-13.3)	9 (4-18)	0.837

Supplemental Table 2C: Baseline characteristics of patients that underwent an endoscopic evaluation at week 52. Data are presented as n (%) or median (IQR). FL-IFX, first-line IFX; wPCDAI, weighted paediatric Crohn's disease activity index; CRP, C-reactive protein; ESR, erythrocyte sedimentation rate; SES-CD, Simple Endoscopic Score for Crohn's Disease; EEN, exclusive enteral nutrition. †perianal disease comprised inactive fistula, skin tags, or anal fissures.

	First-line IFX		p-value	Conventional		P-value
**Received treatment escalation**	**Yes**	**No**		**Yes**	**No**	
**wPCDAI, median (IQR)**	17.5 (0.0-35.0)	0.0 (0.0-22.5)	0.109	21.3 (8.1-31.9)	10 (0.0-20.0)	**0.020**
**Fcal, µg/g, median (IQR)**	312 (193-896)	89 (34-496)	**0.027**	572 (403-219)	139 (93-806)	**0.023**
**CRP, mg/L, median (IQR)**	2.0 (1.1-4.6)	0.7 (0.0-2.6)	**0.003**	10.0 (0.9-26.5)	4.8 (0.9-8.7)	0.277
**ESR, mm/hour, median (IQR)**	12.0 (5.5-24.5)	3.0 (1.0-6.3)	**0.023**	17.0 (2.8-33.8)	10.0 (3.0-17.0)	0.176

Supplemental Table 3: Levels of inflammatory markers after induction treatment in patients that received and did not receive treatment escalation. Levels are depicted per treatment group. wPCDAI scores, CRP levels and ESR levels are obtained 14 weeks after start of induction treatment. Fcal levels were obtained 10 weeks after start of induction treatment. IFX=infliximab; wPCDAI= weighted paediatric Crohn's disease activity index; Fcal=faecal calprotectin level; CRP=C-reactive protein; ESR=erythrocyte sedimentation rate.

	First-line IFX (n=47)	Conventional (n=42)	P-value
**Baseline QOL score, median (IQR)**	59.6 (49.5-72.0) *[n=36]*	61.1 (49.5-70.9) *[n=28]*	0.660
**Week 14 QOL score , median (IQR)**	81.8 (70.7-86.8)* *[n=36]*	77.1 (68.6-85.7)* *[n=27]*	0.297
**Week 52 QOL score, median (IQR)**	80.0 (71.4-88.6)* *[n=31]*	77.5 (66.6-85.5)* *[n=28]*	0.261

Supplemental Table 4: Quality of life assessment based on the IMPACT III questionnaire. The total IMPACT III score ranges from 0 to 100 with a higher score indicating a better quality of life. IFX=infliximab; QOL=quality of life.
*significantly higher compared to the score at baseline (p<0.001)
