# Supplementary material for: First-line treatment with infliximab versus conventional treatment in children with newly diagnosed moderate-to-severe Crohn’s disease: an open-label multicentre randomised controlled trial
Source: Gut. 2020 Dec 31;71(1):34–42. doi: 10.1136/gutjnl-2020-322339 (PMC8666701; doi:10.1136/gutjnl-2020-322339)
Supplement: Supplementary data [file gutjnl-2020-322339supp002.pdf]

Legend Supplemental Figures

Supplemental Figure 1. Box and whisker plot depicting the median wPCDAI and interquartile range per visit. P values were calculated with a Mann-Whitney U test. The median wPCDAI at week 22 was considered statistically significant different between treatment groups. Abbreviations: wPCDAI=weighted pediatric Crohn's disease activity index.
