# Supplementary figures and images for: First-line treatment with infliximab versus conventional treatment in children with newly diagnosed moderate-to-severe Crohn’s disease: an open-label multicentre randomised controlled trial
Source: Gut. 2020 Dec 31;71(1):34–42. doi: 10.1136/gutjnl-2020-322339 (PMC8666701; doi:10.1136/gutjnl-2020-322339)

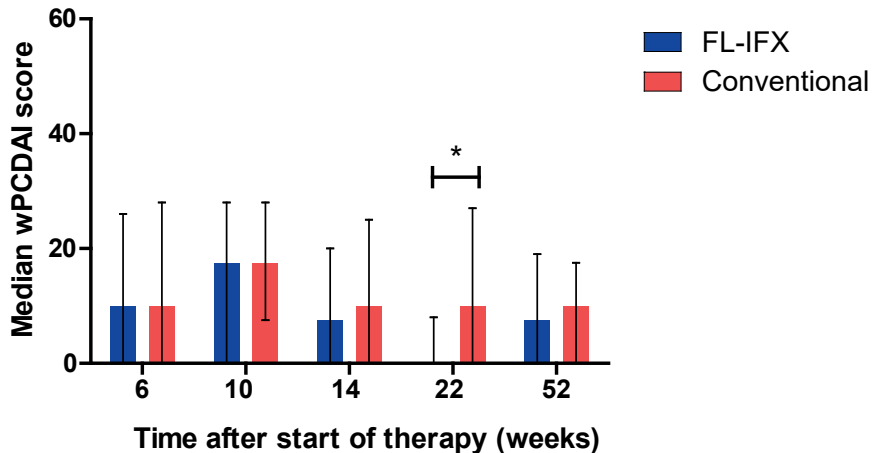

Supplement: Supplementary data [file gutjnl-2020-322339supp003.pdf]
